# Healing the Mind to Ease Pain and Fatigue: The Role of Attachment, Mindfulness, and Cognitive Emotion Regulation in Early‐Stage Breast Cancer Survivors

**DOI:** 10.1002/cnr2.70471

**Published:** 2026-02-28

**Authors:** Kiumars Soleymani, Fatemeh Sadeghi, Rasool Hamidi Choolabi

**Affiliations:** ^1^ Department of Psychology Islamic Azad University Saveh Iran; ^2^ Department of Psychology Islamic Azad University Tehran Iran; ^3^ Department of Mental Health, Mofid Children's Hospital Shahid Beheshti University of Medical Sciences Tehran Iran; ^4^ Department of Psychology Ahrar Institute of Technology and Higher Education Rasht Iran

**Keywords:** attachment styles, breast cancer, cancer‐related fatigue, cognitive emotion regulation strategies, mindfulness skills, pain perception

## Abstract

**Background:**

Pain and cancer‐related fatigue (CRF) are common and debilitating symptoms among breast cancer survivors, significantly impairing quality of life. Psychological factors, including attachment styles, mindfulness skills, and cognitive emotion regulation strategies (CERS), are essential for symptom management.

**Aims:**

This study examined the predictive roles of attachment styles, mindfulness skills, and CERS on pain perception and CRF severity in women with early‐stage breast cancer.

**Methods and Results:**

A descriptive‐correlational design was applied to 201 women recruited from Tehran Shohada Hospital. Participants completed the Adult Attachment Styles (AAS), Kentucky Inventory of Mindfulness Skills (KIMS), Cognitive Emotion Regulation Questionnaire‐Short (CERQ‐Short), McGill Pain Questionnaire (MPQ), and Fatigue Severity Scale (FSS). Regression analyses revealed that attachment styles accounted for 20% of the variance in pain perception and 24% in CRF, with secure attachment reducing and ambivalent attachment exacerbating symptoms. Mindfulness skills explained 45% of pain perception and 26% of CRF variance, with accepting without judgment being the strongest predictor. CERS contributed to 46% of the variance in pain perception and 21% in CRF, with adaptive strategies mitigating and maladaptive strategies amplifying symptoms.

**Conclusions:**

Promoting secure attachment, cultivating mindfulness skills, particularly accepting without judgment, and training adaptive CERS can significantly alleviate pain and fatigue in breast cancer survivors. These findings underscore the value of psychological interventions in enhancing treatment outcomes and quality of life in this population.

## 1 Introduction

Breast cancer remains one of the most pressing global health concerns, representing the most frequently diagnosed malignancy among women worldwide. In 2022 alone, it accounted for over 2.29 million new cases and 666 103 deaths globally [1]. Beyond mortality, the enduring challenges of survivorship include persistent pain and cancer‐related fatigue (CRF), both of which substantially impair daily functioning and quality of life, demonstrating the intricate connection between physical symptoms and psychological health [2]. Moreover, the subjective nature of pain complicates its diagnosis and management [3], while the complexity of cancer treatments further intensifies this challenge [4]. CRF often accompanies pain as a profound and distressing symptom that disrupts both physical and psychological well‐being [5], making their joint management particularly difficult [6].

Beyond these primary symptoms, breast cancer survivors often grapple with broader psychosocial challenges, including altered body image and changes in self‐concept following treatment. Emerging evidence underscores that fostering a positive body image and cultivating self‐compassion can substantially improve psychological adjustment in survivorship. Sebri et al. [7] proposed body‐compassion‐focused interventions that promote more adaptive attitudes toward the post‐cancer body, thereby reducing distress and improving well‐being. Similarly, Burychka et al. [8] emphasized that positive embodiment, self‐compassion, and positive body image act as protective resources against shame and vulnerability, ultimately fostering resilience. These complex challenges highlight the importance of examining psychological mechanisms such as attachment styles [9], mindfulness [10], and cognitive emotion regulation strategies (CERS) [11, 12], which may buffer or exacerbate survivors' experiences of pain, fatigue, and body image concerns [13, 14, 15, 16].

Within this context, attachment styles, developed early in life, emerge as a critical factor shaping how individuals cope with cancer‐related stress. It has evolved as a socio‐emotional perspective that underscores the significance of early infant‐caretaker relationships in an evolutionary‐ecological context for the adaptation and survival of species [17]. Secure attachment fosters emotional stability and resilience [18], whereas insecure attachment can intensify distress, fear, and maladaptive coping [19].

Moreover, mindfulness, defined as the nonjudgmental awareness of the present moment [20], has also demonstrated efficacy in alleviating anxiety, enhancing emotional regulation [21], and reducing both pain [22] and fatigue [23, 24]. Mindfulness skills help patients fully engage with their treatment and cope with the disease's emotional and physical ramifications [21].

Furthermore, CERS, deliberate efforts to manage and reinterpret emotional experiences, are crucial for mitigating the negative psychological impacts [25] that can amplify pain perception [26] and the severity of CRF [27, 28]. By consciously reframing thoughts, reappraising stressors, and consciously altering the interpretation of their experiences, early‐stage breast cancer women may exert a degree of control over their emotional responses [29, 30]. Moreover, these strategies are crucial for cancer survivors' psychological well‐being [31]. Results of a study showed that breast cancer survivors exhibiting adaptive CERS demonstrated reduced fear of cancer recurrence [32]. Recent evidence shows that structured interventions targeting emotion regulation, including cognitive strategies, can significantly improve psychological adjustment among breast cancer survivors, while also influencing body image outcomes [33].

Despite the recognized importance of these psychological dimensions, there is a striking paucity of integrated models in existing research that concurrently assess the effects of attachment styles, mindfulness, and cognitive emotion regulation on pain and fatigue management in breast cancer survivors. Most prior studies have examined these factors in isolation without considering their potential synergistic and cumulative effects. This fragmented perspective limits theoretical understanding and restricts the development of holistic psychological interventions. Our study addresses this gap by adopting a novel integrative framework. We simultaneously examine the predictive roles of attachment styles, mindfulness skills, and CERS in pain perception and CRF among early‐stage breast cancer survivors. The uniqueness of our study lies in its comprehensive approach, combining three theoretically distinct but clinically interrelated domains into a single predictive model. We hypothesize that secure attachment, higher mindfulness skills, and adaptive CERS will predict lower levels of pain and CRF, whereas insecure attachment and maladaptive CERS will predict higher symptom severity. In addition, given the multidimensional nature of these psychological constructs, we also aim to explore which specific sub‐dimensions of attachment, mindfulness, and CERS would emerge as the strongest predictors of pain perception and CRF. This exploratory question allows us to identify the most influential psychological mechanisms for symptom management.

## 2 Method

### 2.1 Study Design and Participants

Our study employed a descriptive‐correlational design and included 201 female breast cancer survivors recruited from the oncology ward of Tehran Shohada Hospital. Recruitment was carried out in collaboration with oncologists and nursing staff, who identified potential participants through hospital records and ongoing follow‐up visits. Eligible women were approached in person during their follow‐up appointments and provided with detailed written and oral information about the study. Participants were selected using convenience and purposive sampling after obtaining ethical approval from the Medical Ethics Committee of Islamic Azad University, Saveh Branch. Of the 305 individuals initially screened for participation, 201 met the eligibility criteria and consented to participate in the study; 8 were excluded due to advanced disease stage or comorbidities, and 96 declined participation after initial contact. A total of 201 participants met the eligibility criteria and consented to participate. During data collection, 5 participants initially withdrew, but replacement recruitment maintained the final analytic sample at 201 women. Thus, the effective dropout rate was negligible. All participants included in the final analysis were in the post‐treatment phase, assessed at least 6 months after completing active cancer treatments such as surgery, chemotherapy, or radiotherapy.

### 2.2 Eligibility Criteria

Participants were required to meet the following inclusion criteria: a confirmed diagnosis of breast cancer by an oncologist, a post‐diagnosis period of at least 6 months, the absence of stage IV cancer, and no significant physical or mental comorbidities. Additionally, participants needed to adhere to their cancer treatment protocols and demonstrate the ability to understand and speak Persian. Informed consent was obtained from all eligible participants after they were provided with detailed information about the study's objectives and procedures. Exclusion criteria included ongoing psychotherapy, the use of psychotropic drugs, or withdrawal from the study at any point during data collection.

### 2.3 Measures

#### 2.3.1 The Adult Attachment Style (AAS) Questionnaire

Developed by Hazan and Shaver in 1987, the Adult Attachment Style (AAS) questionnaire is a self‐report instrument designed to assess adult attachment styles. It consists of 15 items scored on a 7‐point Likert‐type scale, divided into three subscales representing secure, ambivalent, and avoidant attachment styles [34]. The questionnaire has demonstrated satisfactory internal consistency with alpha coefficients ranging from 0.72 to 0.79 [35] and confirmed reliability within the Iranian population [36]. In our study, the subscales exhibited strong internal consistency, with Cronbach's alpha values ranging from 0.73 to 0.80.

#### 2.3.2 The Kentucky Inventory of Mindfulness Skills (KIMS)

The Kentucky Inventory of Mindfulness Skills (KIMS), created by Baer, Smith, and Allen in 2004, is a self‐report measure that evaluates four aspects of mindfulness: observing, describing, acting with awareness, and accepting without judgment. This 39‐item instrument uses a 5‐point Likert scale to gauge how frequently these mindfulness skills are employed, from very rare to most common. Previous studies have noted high internal consistency for the KIMS (*α* = 0.83–0.91) [37], and its reliability has been validated in an Iranian context (*α* = 0.82) [38]. Our study found strong internal consistency for its subscales (*α* = 0.80).

#### 2.3.3 The Cognitive Emotion Regulation Questionnaire‐Short (CERQ‐Short)

The Cognitive Emotion Regulation Questionnaire‐Short (CERQ‐Short) is an 18‐item derivative of the original CERQ developed by Garnefski and Kraaij in 2006. This questionnaire assesses how individuals cognitively manage stressful or threatening events across nine subscales, each represented by two items. It employs a 5‐point Likert scale (ranging from 1 to 5), with higher scores indicating a greater use of specific cognitive strategies [39]. The CERQ‐Short has shown good psychometric properties in its original and Persian versions, with Cronbach's alpha values ranging from 0.68 to 0.82 [40]. Our study reported strong internal consistency for its subscales (*α* = 0.68 to 0.80).

#### 2.3.4 The McGill Pain Questionnaire (MPQ)

Developed by Melzack in 1975, the McGill Pain Questionnaire (MPQ) is a comprehensive tool designed to measure pain perception. It features 20 sets of items that evaluate pain across sensory (sections 1–10), affective (sections 11–15), and evaluative dimensions (section 16), alongside miscellaneous items (sections 17–20). This instrument has been validated extensively, including within the Iranian population, demonstrating Cronbach's alpha values between 0.81 and 0.96 [41]. In this study, the MPQ showed strong internal consistency for its pain components (*α* = 0.80–0.90).

#### 2.3.5 The Fatigue Severity Scale (FSS)

Developed by Krupp et al. in 1989, the Fatigue Severity Scale (FSS) consists of 9 items derived from a larger 28‐item fatigue scale. This scale is scored on a Likert scale ranging from 1 to 7, designed to measure an individual's perceptions of fatigue severity and its impact on their daily functioning. The scoring system categorizes fatigue levels: low fatigue, which ranges from 9 to 18; moderate fatigue, spanning scores from 18 to 45; and high fatigue, indicated by scores above 45 [42]. This scale has demonstrated excellent psychometric properties, including optimal validity and reliability within Iranian populations, where it achieved a Cronbach's alpha of 0.96 [43]. The FSS has demonstrated excellent internal consistency in our study (*α* = 0.90), confirming its reliability for evaluating fatigue severity among breast cancer survivors.

### 2.4 Procedure

Data collection was conducted over 1 month in the oncology department of Tehran Shohada Hospital. All participants were assessed at least 6 months after completing active cancer treatments, including surgery, chemotherapy, or radiotherapy. This post‐treatment phase ensured a uniform baseline for evaluating psychological factors and their relationship with pain perception and CRF. Participants completed self‐report assessments under the supervision of a trained researcher. For those with physical limitations, such as impaired vision or difficulty writing, the researcher provided assistance by reading the items aloud and recording responses verbatim. Ethical considerations were strictly observed, with informed consent obtained prior to participation.

### 2.5 Statistical Analysis

Data were analyzed using SPSS version 24. Descriptive statistics (mean, standard deviation, skewness, and kurtosis) confirmed normality for all variables. Pearson correlation coefficients were calculated to assess relationships between psychological constructs (attachment styles, mindfulness skills, and CERS) and outcomes (pain perception and CRF). Moreover, multiple linear regression analyses were conducted to identify predictors of pain perception and CRF. Independent variables included attachment styles (secure, avoidant, ambivalent), mindfulness skills (observing, describing, acting with awareness, and accepting without judgment), and CERS (adaptive, maladaptive). Regression assumptions, including normality of residuals and multicollinearity (variance inflation factor < 10), were satisfied. Adjusted *R* ^2^ values, standardized beta coefficients (*β*), and significance levels (*p* < 0.05) were reported.

## 3 Results

### 3.1 Descriptive Statistics

Table 1 summarizes participants' demographic and clinical characteristics. The mean age was 49.0 years (SD = 8.0); 39.8% were employed. Most had received radiotherapy (71.1%), and 41.3% had undergone chemotherapy. Regarding cancer stage, 17.9% were stage I, 57.2% stage II, and 24.9% stage III. These variables were statistically controlled in regression models; none reached significance (all *p*s > 0.1), indicating no effects on pain or CRF. Detailed regression results for these covariates are available in Appendix S1.

**TABLE 1:** Demographic and clinical characteristics of participants (*n* = 201).

Variables	Category	*n*	%
1. Age (years)	M ± SD	49.0 ± 8.0	—
2. Employment status	Employed	80	39.8
	Unemployed	121	60.2
3. Chemotherapy	Yes	83	41.3
	No	118	58.7
4. Radiotherapy	Yes	143	71.1
	No	58	28.9
5. Cancer stage	I	36	17.9
	II	115	57.2
	III	50	24.9

Abbreviations: M, mean; SD, standard deviation.

Descriptive statistics for the study variables, including means, standard deviations, skewness, and kurtosis, are presented in Table 2. CRF had a mean score of 38.01 (SD = 9.59), while pain perception averaged 89.09 (SD = 16). Skewness and kurtosis values for all variables were within acceptable ranges (±1.96), indicating normal data distributions suitable for parametric analyses.

**TABLE 2:** Descriptive statistics and distribution measures for study variables (*n* = 201).

Variables	*M*	SD	Skewness	Kurtosis
1. Pain perception	89.09	16	−0.72	0.85
2. CRF	38.01	9.59	0.74	0.15
3. Secure	18.51	5.63	−0.22	−0.52
4. Avoidant	11.82	7.07	0.75	−0.71
5. Ambivalent	2.44	1.25	0.52	−0.65
6. Observing	12.59	4.50	0.76	−0.75
7. Describing	23.53	7.23	−0.20	−0.39
8. Acting with awareness	17.89	6.06	−0.36	−0.35
9. Accepting without judgment	18.64	5.49	−0.61	0.44
10. Adaptive CERS	11.67	3.59	−0.02	0.09
11. Maladaptive CERS	15.17	3.66	−0.50	−0.88

Abbreviations: CERS, cognitive emotion regulation strategies; CRF, cancer‐related fatigue; M, mean; SD, standard deviation.

### 3.2 Correlation Analysis

The correlation analysis revealed significant relationships between psychological constructs and the outcomes of pain perception and CRF, highlighting the interplay of attachment styles, mindfulness skills, and CERS in symptom severity. Pain perception is negatively correlated with secure attachment style (*r* = −0.37, *p* < 0.01) and positively correlated with avoidant (*r* = 0.40, *p* < 0.01) and ambivalent attachment styles (*r* = 0.42, *p* < 0.01). Mindfulness skills demonstrated similar trends, where describing (*r* = −0.56, *p* < 0.01) and accepting without judgment (*r* = −0.60, *p* < 0.01) showed the strongest negative correlation with pain perception. Moreover, adaptive CERS were found to have a strong negative correlation with pain perception (*r* = −0.67, *p* < 0.01). In contrast, maladaptive strategies have a positive correlation with pain perception (*r* = 0.42, *p* < 0.01) (see Table 3).

**TABLE 3:** Pearson correlation matrix for study variables (*n* = 201).

Variables	1	2	3	4	5	6	7	8	9	10	11
1. Pain perception	1										
2. CRF	0.32*	1									
3. Secure	−0.37*	−0.47*	1								
4. Avoidant	0.40*	0.38*	−0.55*	1							
5. Ambivalent	0.42*	0.38*	−0.52*	0.87*	1						
6. Observing	−0.37*	−0.38*	0.82*	−0.59*	−0.60*	1					
7. Describing	−0.56*	−0.48*	0.50*	−0.61*	−0.57*	0.41*	1				
8. Acting with awareness	−0.36*	−0.35*	0.64*	−0.46*	−0.48*	0.70*	0.36*	1			
9. Accepting without judgment	−0.60*	−0.43*	0.38*	−0.29*	−0.33*	0.39*	0.46*	0.55*	1		
10. Adaptive CERS	−0.67*	−0.40*	0.51*	−0.41*	−0.41*	0.43*	0.76*	0.41*	0.37*	1	
11. Maladaptive CERS	0.42*	0.39*	−0.68*	0.49*	0.51*	−0.70*	−0.43*	−0.39*	−0.46*	−0.46*	1

Abbreviations: CERS, cognitive emotion regulation strategies; CRF, cancer‐related fatigue.

* *p* ≥ 0.01.

Pearson correlation analyses also revealed significant associations between CRF and the examined psychological constructs. CRF negatively correlated with secure attachment style (*r* = −0.47, *p* < 0.01) and positively with both avoidant (*r* = 0.38, *p* < 0.01) and ambivalent attachment styles (*r* = 0.38, *p* < 0.01). Mindfulness skills including observing (*r* = −0.38, *p* < 0.01), describing (*r* = −0.48, *p* < 0.01), acting with awareness (*r* = −0.35, *p* < 0.01), and accepting without judgment (*r* = −0.43, *p* < 0.01) all showed significant negative correlations with CRF. Similarly, adaptive CERS were negatively correlated with CRF (*r* = −0.40, *p* < 0.01), while maladaptive strategies were positively correlated (*r* = 0.39, *p* < 0.01) (see Table 3).

### 3.3 Regression Analysis

Multiple regression analyses were conducted to predict pain perception and CRF. Secure attachment style (*β* = −0.21, *t* = −2.73, *p* = 0.007) and avoidant attachment style (*β* = 0.29, *t* = 2.21, *p* = 0.028) explained 20% of the variance in pain perception. Accepting without judgment emerged as a strong predictor, alone accounting for 45% of the variance in pain perception (*β* = −0.55, *t* = −6.58, *p* < 0.001). Furthermore, adaptive (*β* = −0.61, *t* = −10.35, *p* < 0.001) and maladaptive CERS (*β* = 0.14, *t* = 2.39, *p* = 0.018) collectively accounted for 46% of the variance in pain perception (see Table 4). Regarding CRF, secure attachment style was a significant predictor, explaining 24% of the variance (*β* = −0.37, *t* = −5.03, *p* < 0.001). Describing mindfulness skills also accounted for 26% of the variance in CRF (*β* = −0.37, *t* = −3.89, *p* < 0.001). Together, adaptive (*β* = −0.28, *t* = −3.93, *p* < 0.001) and maladaptive CERS (*β* = 0.27, *t* = 3.78, *p* < 0.001) explained 21% of the variance in CRF (see Table 5). These findings underscore the complex interplay between psychological variables and both pain perception and CRF in early‐stage breast cancer survivors.

**TABLE 4:** Regression analysis summary for predictors of pain perception.

Effects	B	SE	*β*	*t*	Sig	*F*	adj *R* ^2^
Constant	20.79	2.19		9.49	0.000	17.96*	0.20
Secure	−0.18	0.07	−0.21	−2.73	0.007		
Avoidant	0.03	0.11	0.04	0.28	0.78		
Ambivalent	0.26	0.12	0.29	2.21	0.028		
Constant	34.17	1.03		33.20	0.000	42.82*	0.45
Observing	−0.04	0.05	−0.05	−0.69	0.49		
Describing	−0.61	0.49	−0.10	−1.24	0.22		
Acting with awareness	−0.07	0.08	−0.06	−0.78	0.43		
Accepting without judgment	−0.38	0.06	−0.55	−6.58	0.000		
Constant	29.83	1.90		15.72	0.000	87.01*	0.46
Adaptive CERS	−0.85	0.08	−0.61	−10.35	0.000		
Maladaptive CERS	0.19	0.08	0.14	2.39	0.018		

Abbreviation: CERS, cognitive emotion regulation strategies.

* *p* ≥ 0.01.

**TABLE 5:** Regression analysis summary for predictors of CRF.

Effects	B	SE	*β*	*t*	Sig	*F*	adj *R* ^2^
Constant	15.62	2.61		5.98	0.000	22.11*	0.24
Secure	−0.39	0.08	−0.37	−5.03	0.000		
Avoidant	0.08	0.13	0.08	0.62	0.54		
Ambivalent	0.13	0.14	0.11	0.91	0.36		
Constant	20.60	1.47		14.06	0.000	18.81*	0.26
Observing	−0.14	0.08	−0.16	−1.79	0.07		
Describing	−2.72	0.70	−0.37	−3.89	0.000		
Acting with awareness	−0.13	0.12	−0.10	−1.11	0.27		
Accepting without judgment	−0.01	0.08	−0.01	−0.13	0.90		
Constant	10.76	2.81		3.83	0.000	27.87*	0.21
Adaptive CERS	−0.48	0.12	−0.28	−3.93	0.000		
Maladaptive CERS	0.45	0.12	0.27	3.78	0.000		

Abbreviation: CERS, cognitive emotion regulation strategies.

* *p* ≥ 0.01.

## 4 Discussion

Our study aimed to investigate the predictive role of attachment styles, mindfulness skills, and CERS in modulating pain perception and CRF among women with early‐stage breast cancer. We observed that these psychological constructs interact dynamically to shape the experience of pain and fatigue, offering critical insights into the biopsychosocial mechanisms underlying chronic symptoms in this population. Adaptive CERS emerged as the strongest predictor of pain perception, followed by the mindfulness component of accepting without judgment, consistent with studies emphasizing the effectiveness of mindfulness‐based interventions in reducing cancer‐related distress [44, 45] and physical symptoms [46]. Conversely, maladaptive strategies were strongly associated with increased pain perception, aligning with findings from Rogers et al., which showed that maladaptive cognitive strategies heightened pain intensity, interference, and negative affect in chronic pain patients [47].

Secure attachment also played a protective role, while ambivalent attachment was associated with higher pain levels. This aligns with evidence showing that secure attachment fosters emotional stability and enhances the use of social support, while ambivalent attachment heightens emotional distress and maladaptive coping [48]. Recent reviews and empirical studies further confirm that insecure attachment, particularly attachment anxiety, is associated with a greater risk of persistent post‐treatment pain and psychological distress, whereas secure attachment facilitates resilience and adaptive coping in breast cancer patients [49, 50, 51].

In the case of CRF, secure attachment was the most significant predictor, emphasizing the importance of emotional trust and social support. These findings align with evidence suggesting that secure attachment enhances psychological resilience, which contributes to better coping mechanisms during cancer treatment [52]. Resilience, fostered by secure attachment, likely reduces the psychological burden of CRF and facilitates better symptom management. Furthermore, adaptive CERS provide additional resilience by enabling patients to reinterpret stressors in a less distressing manner, which has been shown to mitigate fatigue and improve overall quality of life. Among mindfulness components, describing emerged as a key predictor of CRF reduction, reflecting its role in addressing the cognitive aspects of fatigue, a finding that contrasts with studies suggesting mindfulness components like observing may play a broader role [53]. Evidence from network analysis and meta‐analyses further underscores that mindfulness traits (especially accepting without judgment and describing) are central nodes in improving emotional functioning and quality of life in breast cancer survivors, highlighting their role in reducing both fatigue and negative affect [54, 55, 56].

Attachment theory offers a strong framework to explain how these psychological constructs interact to influence health outcomes [57, 58]. The impact of internalized attachment styles is profound, affecting how individuals interact with their environment and respond to health‐related challenges [19]. Secure attachment enhances emotional security and fosters robust social support networks [48], which are critical in mitigating pain [59] and CRF [60, 61]. These secure connections provide stability and enable individuals to cope effectively with the challenges of chronic illness [62]. Conversely, insecure attachment styles, particularly ambivalent attachment, increase emotional distress and maladaptive coping, which intensify symptoms [48]. This perspective is further strengthened by findings that attachment security directly predicts lower symptom burden and indirectly improves adjustment through enhanced social connectedness [49, 50].

Mindfulness, defined as the practice of nonjudgmental awareness and acceptance of one's moment‐to‐moment experiences, enables individuals to address distressing thoughts and feelings directly rather than avoiding them [20]. This adaptive approach disrupts the cycle of pain and psychological distress by preventing automatic negative thoughts from becoming entrenched, allowing for more constructive and positive reevaluation of painful experiences [54]. As demonstrated in our study, mindfulness not only reshapes the perception of pain and fatigue but also strengthens individuals' capacity to manage these symptoms effectively [21, 22, 23, 24]. The differential effects of mindfulness components, such as accepting without judgment for pain and describing for fatigue, highlight the nuanced ways mindfulness can target specific symptoms. Recent longitudinal and intervention studies confirm that mindfulness‐based stress reduction significantly decreases perceived stress, anxiety, depression, and fear of recurrence, thereby enhancing overall quality of life in breast cancer survivors [56].

Furthermore, our study highlighted the critical role of CERS in pain and CRF management, pinpointing specific areas for psychological intervention. Adaptive CERS, such as positive reappraisal, can decouple the physical sensation of pain from its emotional impact, reducing the overall experience of pain and enhancing quality of life. Conversely, maladaptive strategies can exacerbate pain perception and CRF and contribute to poorer health outcomes [26, 27, 28, 63]. Recent evidence supports this mechanism: adaptive strategies like positive refocusing and reappraisal buffer the impact of anxiety on fatigue, whereas maladaptive strategies such as rumination, catastrophizing, or self‐blame exacerbate fatigue severity and lower quality of life in cancer survivors [64, 65].

These findings demonstrate the complex interplay of attachment styles, mindfulness skills, and CERS in shaping pain and fatigue outcomes. By incorporating these insights into integrative interventions, it is possible to address both symptoms holistically. Such approaches can alleviate the dual burden of pain and fatigue and ultimately enhance the quality of life for breast cancer survivors.

While our study provides valuable insights, several limitations should be acknowledged. The modest sample size and recruitment from a single medical center restrict the generalizability of the findings, reducing their applicability to more diverse populations. The cross‐sectional design further limits our ability to infer causality, as it captures a correlational snapshot at a single time point rather than dynamic changes over time. Additionally, potential confounders, such as hormonal treatments, comorbidities, and medication usage, as well as sociodemographic variables like marital status and education level, were not fully accounted for, which may have influenced the observed relationships between psychological constructs and outcomes related to pain and fatigue. Moreover, the study's focus on early‐stage female breast cancer patients further narrows its scope by excluding male patients and individuals at other stages of breast cancer. These exclusions limit the applicability of the findings to the broader cancer population. Addressing these limitations would strengthen the evidence base and enhance the relevance of the results to diverse clinical settings.

To enhance the robustness of future studies, it is essential to address these limitations. Increasing the sample size, recruiting from multiple centers, and including male patients and diverse cancer stages will improve the representativeness and generalizability of findings. Future research should also incorporate a wider range of clinical and sociodemographic covariates to better capture potential confounding effects and clarify their impact on pain and fatigue. Employing longitudinal or experimental designs will help establish causal pathways, offering deeper insights into the psychological mechanisms influencing pain and fatigue, and enabling the development of more targeted interventions for breast cancer survivors.

In conclusion, our study substantiates the crucial roles of attachment styles, mindfulness skills, and CERS in modulating pain perception and CRF among early‐stage breast cancer survivors. The robust predictive role of secure attachment emphasizes the protective effects of emotional security and strong social support networks in mitigating the dual burdens of pain and fatigue. Mindfulness components demonstrated nuanced impacts on symptom management, with accepting without judgment emerging as particularly effective in reducing pain by fostering nonreactive awareness, and describing showing greater relevance for alleviating fatigue through emotional articulation. Moreover, CERS, particularly adaptive ones like positive reappraisal, played a pivotal role in reducing the intensity of both pain and fatigue. In contrast, maladaptive strategies were associated with symptom exacerbation, underscoring the importance of integrating training in adaptive CERS into breast cancer care. Together with converging evidence from recent empirical and review studies [49, 50, 54, 56, 64], these findings advocate for holistic treatment approaches that address not only the physical but also the emotional and psychological well‐being of breast cancer survivors.

## Author Contributions

**Kiumars Soleymani:** conceptualization, methodology, data curation, formal analysis, writing – original draft, software. **Fatemeh Sadeghi:** supervision, project administration, resources, validation, formal analysis. **Rasool Hamidi Choolabi:** writing – review and editing, visualization, investigation, validation.

## Funding

The authors have nothing to report.

## Ethics Statement

The Medical Ethics Committee of Islamic Azad University, Saveh Branch, Saveh, Iran approved the study protocol.

## Consent

Informed consent was obtained from all participants included in this study. The participants were fully informed about the nature, purpose, and potential risks of the research, and their rights to withdraw at any stage without consequence were emphasized. All data were anonymized to protect participant privacy and confidentiality.

## Conflicts of Interest

The authors declare no conflicts of interest.

## Supporting information


**Appendix S1:** contains the regression analyses results examining the predictive power of demographic and clinical covariates (Age, Employment, Chemotherapy, Radiotherapy, and Cancer Stage) on both pain perception and CRF scores among the study sample (*N* = 201).

## Data Availability

The data that support the findings of this study are available from the corresponding author upon reasonable request.
