# Supplementary material for: Healing the Mind to Ease Pain and Fatigue: The Role of Attachment, Mindfulness, and Cognitive Emotion Regulation in Early‐Stage Breast Cancer Survivors
Source: Cancer Rep (Hoboken). 2026 Feb 28;9(3):e70471. doi: 10.1002/cnr2.70471 (PMC12949395; doi:10.1002/cnr2.70471)
Supplement: Supplementary file 1 — Appendix S1: contains the regression analyses results examining the predictive power of demographic and clinical covariates (Age, Employment, Chemotherapy, Radiotherapy, and Cancer Stage) on both pain perception and CRF scores among the study sample (N = 201). [file CNR2-9-e70471-s001.docx]

Appendix S1 contains the regression analyses results examining the predictive power of demographic and clinical covariates (Age, Employment, Chemotherapy, Radiotherapy, and Cancer Stage) on both pain perception and CRF scores among the study sample (N = 201).

| **Predictor** | **B (Pain)** | **SE (Pain)** | **β (Pain)** | ***p* (Pain)** | **B (CRF)** | **SE (CRF)** | **β (CRF)** | ***p* (CRF)** |
| --- | --- | --- | --- | --- | --- | --- | --- | --- |
| Age | 0.05 | 0.06 | 0.07 | 0.38 | 0.02 | 0.04 | 0.06 | 0.63 |
| Employment | 0.42 | 1.20 | 0.03 | 0.72 | 0.25 | 0.51 | 0.04 | 0.63 |
| Chemotherapy | 0.60 | 1.08 | 0.05 | 0.58 | 0.18 | 0.38 | 0.03 | 0.62 |
| Radiotherapy | 0.72 | 1.45 | 0.04 | 0.62 | 0.12 | 0.49 | 0.02 | 0.80 |
| Stage II | 0.18 | 1.98 | 0.01 | 0.98 | 0.58 | 0.62 | 0.09 | 0.35 |
| Stage III | 1.23 | 1.56 | 0.08 | 0.43 | 0.74 | 0.75 | 0.10 | 0.33 |

**Appendix S1** Regression results for demographic and clinical covariates predicting pain perception and CRF (N = 201)

Note. Stage I was used as the reference category.
